# Diversity of piroplasmids in wild animals in Brazil: a review

**DOI:** 10.1590/S1984-29612025061

**Published:** 2025-10-27

**Authors:** Ana Cláudia Calchi, João Fabio Soares, Rosangela Zacarias Machado, Marcos Rogério André

**Affiliations:** 1 Universidade Estadual Paulista “Júlio de Mesquita Filho” – UNESP, Faculdade de Ciências Agrárias e Veterinárias – FCAV, Departamento de Patologia, Reprodução e Saúde Única, Vector-Borne Bioagents Laboratory – VBBL, Jaboticabal, SP, Brasil; 2 Universidade Federal do Rio Grande do Sul – UFRGS, Faculdade de Veterinária, Departamento de Patologia e Clínica Veterinária, Porto Alegre, RS, Brasil

**Keywords:** *Babesia* spp., *Theileria* spp., *Cytauxzoon* spp., wildlife, phylogeny, *Babesia* spp., *Theileria* spp., *Cytauxzoon* spp., fauna selvagem, filogenia

## Abstract

The study of the diversity of tick-borne agents in wild animals enables a better understanding of the distribution of pathogens in the country, the identification of potential reservoirs for these agents, the mapping of possible infection hotspots, the implementation of local fauna management, and the development of species conservation strategies, as well as the creation of disease control and prevention strategies. Piroplasmids are apicomplexan protozoa that primarily infect blood cells of vertebrates and can cause diseases in animals and humans. In Brazil, several studies have identified a wide diversity of piroplasmids in different species of wild animals. This review article aims to compile these studies, with an emphasis on the species detected according to host orders and families, the diagnostic methods used, the occurrence of clinical signs, and the distribution of these agents across the country. It also aims to review the phylogenetic topology of piroplasmids by including the new species and clades detected in the country.

## Resumo

O estudo da diversidade de agentes transmitidos por carrapatos em animais selvagens possibilita um melhor entendimento da distribuição de patógenos no país, a identificação de possíveis reservatórios para esses agentes, o mapeamento de potenciais focos de infecção, a atuação no manejo das faunas locais e a formulação de estratégias de conservação das espécies, além da criação de estratégias de controle e prevenção de doenças. Piroplasmídeos são protozoários apicomplexas que infectam principalmente células sanguíneas de vertebrados, podendo causar doenças em animais e humanos. No Brasil, diversos estudos têm identificado uma ampla diversidade de piroplasmídeos em diferentes espécies de animais selvagens. Este artigo de revisão compila esses estudos, com ênfase nas espécies detectadas de acordo com as ordens e famílias de hospedeiros, nos métodos de diagnóstico utilizados, na ocorrência de sinais clínicos e na distribuição desses agentes no país. Adicionalmente, revisa a topologia da filogenia de piroplasmídeos por meio da inclusão das novas espécies e clados detectados no país.

## Introduction

Piroplasmids (Apicomplexa: Piroplasmorida) constitute the second most prevalent group of hemoparasites in mammals, surpassed only by *Trypanosoma* spp. (Alvarado-Rybak et al., 2016; Schnittger et al., 2022). These apicomplexan protozoa are classified within the domain *Eukarya*, supergroup Diaphoretickes, clade TSAR, group SAR, clade Alveolata, phylum Apicomplexa, class Aconoidasida, and order Piroplasmorida (Adl et al., 2019; Burki et al., 2020). The order comprises the families Babesiidae—including the genera *Babesia* and *Rangelia*—and Theileriidae, which includes *Theileria* and *Cytauxzoon* (Solano-Gallego & Baneth, 2011; Schnittger et al., 2012; Alvarado-Rybak et al., 2016).

These protozoa infect a variety of vertebrate blood cells, such as erythrocytes, lymphocytes, and monocytes, as well as endothelial cells, depending on the species involved. These parasites are etiological agents of diseases affecting both domestic and wild animals, and in some cases, humans (Alvarado-Rybak et al., 2016; Krause, 2019; Schnittger et al., 2022).

Babesiosis is considered an emerging zoonosis, with reported cases across North America, South America, Europe, Asia, and Australia (Krause, 2019; Kumar et al., 2021; Karshima et al., 2022). The *Babesia* species identified to date as infecting humans include *B. microti* (including *B. microti*-like), *B. duncani*, *B. divergens* (including *B. divergens*-like MO1), *B. venatorum*, *B. motasi*-like, *Babesia crassa* (including *B. crassa*-like), *B. bigemina*, and *B. odocoilei* (Herwaldt et al., 2004; Yabsley & Shock, 2013; Arsuaga et al., 2016; Chen et al., 2019; Gray & Herwaldt, 2019; Hong et al., 2019; Kumar et al., 2021; Scott et al., 2021; Doderer-Lang et al., 2022; Karshima et al., 2022; Maggi et al., 2024).

In Brazil, cases of symptomatic human babesiosis (characterized by fever, inappetence, and body aches) were reported in Pernambuco based on blood smear analysis, although the species involved was not identified. Asymptomatic cases were also reported in Rio Grande do Sul, where *B. microti* was hypothesized as the probable causative agent (Alecrim et al., 1983; Rech et al., 2004). Additionally, antibodies against *B. bovis* were detected in patients with Baggio-Yoshinari Syndrome (a Lyme-like disease) in the state of São Paulo, although serological cross-reactions could not be ruled out (Yoshinari et al., 2003).

Transmission of piroplasmids occurs primarily through the bite of ixodid ticks, which inoculates the pathogen via their saliva (Jalovecka et al., 2019). Nonetheless, several alternative, albeit less common, transmission routes have been documented. These include blood transfusion, reported in humans infected with *Babesia microti*, *Babesia duncani*, and *Babesia divergens*-like species, as well as in dogs infected with *Babesia gibsoni* (Stegeman et al., 2003; Tang & Tran, 2020; Drews et al., 2023). Transplacental transmission has also been described in multiple host species: in dogs (*Babesia vogeli* and *B. gibsoni*), horses (*Theileria equi*), cattle (*Babesia bovis* and *Babesia bigemina*), and humans (*B. microti*) (Corrêa, 1974; Fukumoto et al., 2005; Krause & Vannier, 2012; Costa et al., 2016; Sant et al., 2016; Agrawal et al., 2020; Rojas-Pirela et al., 2021; Bonato et al., 2023). Iatrogenic transmission through contaminated needles or medical instruments has also been reported (Scoles & Ueti, 2015; Karasová et al., 2022; Schnittger et al., 2022).

Additionally, direct transmission of *B. gibsoni* between dogs has been suspected to occur via aggressive interactions, particularly through bite wounds (Birkenheuer et al., 1999; Matsuu et al., 2004; Jefferies et al., 2007). An experimental study by Corduneanu et al. (2020) detected *Babesia canis* DNA in the organs of mice that had ingested *Dermacentor reticulatus* ticks infected with the parasite, suggesting the possibility of oral transmission as an alternative route of infection.

The diagnosis can be confirmed through microscopic examination of peripheral blood smears. However, the sensitivity of this method is highly dependent on the level of parasitemia and the stage of infection, as these factors directly influence the number of infected cells present in the bloodstream. In addition, this technique requires a skilled person to ensure a more accurate diagnosis (Huber et al., 2017; Momčilović et al., 2019; Kumar et al., 2022). Serological assays represent another diagnostic approach, but they are limited by their inability to confirm active infection and by the potential for cross-reactivity among different *Babesia* or *Theileria* species (Todorovic, 1975; Herwaldt et al., 2003; Kukina et al., 2018; Kumar et al., 2022). Currently, molecular diagnostic methods—particularly polymerase chain reaction (PCR)-based assays—are widely employed due to their superior sensitivity and specificity (Huber et al., 2017; Momčilović et al., 2019; Calchi et al., 2025a).

In recent years, several novel species of piroplasmids have been described. These newly identified lineages are frequently associated with specific vertebrate host taxa, supporting the hypothesis of co-speciation between parasites and hosts. Nevertheless, some piroplasmid species exhibit the capacity to infect multiple host species. This phenomenon is particularly evident within the *Babesia* sensu stricto group, in which the ability of tick vectors to transmit the parasite transovarially facilitates host switching, even across different vertebrate species (Jalovecka et al., 2019).

Although less common, similar host-switching events have been observed in other piroplasmid lineages. However, for phylogenetic groups that rely exclusively on transstadial transmission (i.e., transfer between life stages within the same individual tick), the capacity to infect a broader host range is more limited—unless the tick vector itself is heteroxenic, capable of feeding on multiple host species throughout its life cycle (Jalovecka et al., 2019).

In Brazil, a growing number of genotypes and novel piroplasmid species have been identified in a wide range of wild animal hosts. The aim of this study was to conduct a comprehensive review of the piroplasmid infections reported in wild animals in Brazil. This includes compiling and analyzing data on the species detected to date, their genetic diversity, geographic distribution, and host range. Furthermore, we propose an updated framework for the phylogenetic classification of piroplasmids, building upon the clade structure previously defined by Jalovecka et al. (2019) and incorporating novel species and lineages identified in Brazil.

## Piroplasmids in Wild Animals from Brazil

**Figure 1:**
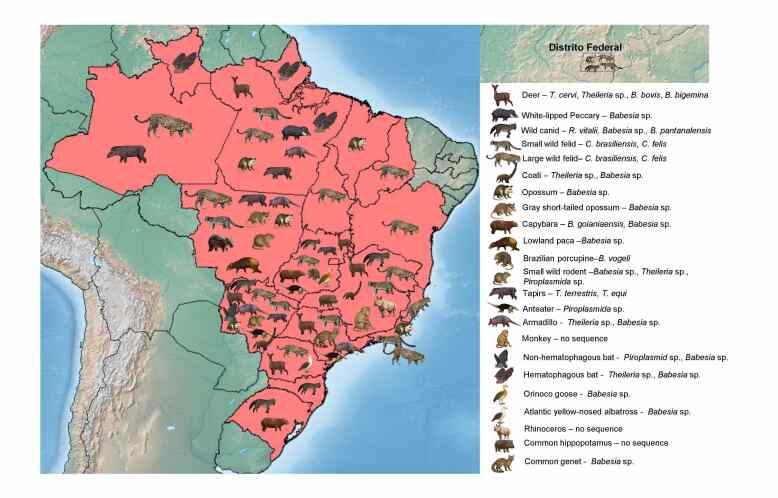
Map showing the distribution, host groups, and piroplasmid species detected in each group across Brazil. Only studies that reported sampling locations for each individual animal were included.

Table 1 summarizes all reports of piroplasmids in wild animals in Brazil, including the diagnostic method and type of sample used, phylogenetic results when available, the locations where positive samples were collected, and whether clinical signs were observed. Figure 1 illustrates the distribution of these studies across the country.

**Table 1:** Studies conducted in Brazil on the detection and occurrence of piroplasmids in wild animals in the country.

**Order**	**Family**	**Host species**	**Technique (Target gene)**	**Size of the obtained sequences or amplified fragment (bp)**	**Type of sample analyzed and positive in screening**	**No. of animals positive in screening / No. of animals analyzed (%)**	**Piroplasmid species and/or phylogenetic placement.**	**Sample origin – Brazilian state (Captivity-C or Free-ranging-FR)**	**Clinical signs and/or lesions**	**Reference**
**Mammals**										
Artiodactyla	Cervidae	*Blastocerus dichotomus* (Marsh deer)	nPCR (18S rRNA)	360 to 420	Blood	2/4 (50%)	*T. cervi* (n=1) *B. bovis* (n=1)	MG (C)	No	Silveira et al. (2011)
			nPCR (18S rRNA), cPCR (18S rRNA, *cox-3*, *hsp70*)	~1120 to 1500 – 18S rRNA~650 – *hsp70* 572 to 650 – *cox-3*	Buffy coat	101/138 (73.2%)	*Theileria* sp. (“*Theileria* sensu stricto” clade)	MS (n=83) and SP (n=18)	-	Calchi et al. (2024a)
		*Mazama americana* (red brocket deer)	cPCR (18S rRNA)	434	Lung and liver	2/2	*Theileria* sp. phylogenetically related to *T. cervi*	PA (FR)	**-**	Soares et al. (2017)
			cPCR (18S rRNA)	551	Blood	1/1	No sequence	GO (FR)	No	Bittencourt et al. (2025)
		*Mazama jucunda* (small red brocket deer)	nPCR (18S rRNA), cPCR (18S rRNA, *hsp70*)	1590 – 18S rRNA 599 to 620 – *hsp70*	Buffy coat	4/4	*Theileria* sp. (“*Theileria* sensu stricto” clade)	SP (FR)	-	Calchi et al. (2024a)
		*Mazama rufa* (southern red brocket deer)	nPCR (18S rRNA), cPCR (18S rRNA, *hsp70*)	1590 – 18S rRNA 599 to 620 – *hsp70*	Buffy coat	3/3	*Theileria* sp. (“*Theileria* sensu stricto” clade)	PR (FR)	-	Calchi et al. (2024a)
		*Ozotoceros bezoarticus* (Pampas deer)	nPCR (18S rRNA)	360 to 450	Blood	23/60 (38.3%)	*T. cervi* (n=12) *B. bovis* (n=3) *B. bigemina* (n=2)	MS (FR)	**-**	Silveira et al. (2013)
			cPCR	1684	Blood	1/1	No sequence	GO and/or MG (C)	No	Fava et al. (2022)
			nPCR (18S rRNA), cPCR (18S rRNA, *hsp70*)	-	Buffy coat	6/10 (60%)	No sequence	MS (FR)	-	Calchi et al. (2024a)
		*Subulo gouazoubira* (gray brocket deer)	Blood smear and nPCR (18S rRNA)	360 to 420	Blood	7/12 (58.3%) – blood smear 13/17 (76.5%) - PCR	*T. cervi* (n=9) *Theileria* sp. (n=3) *B. bigemina* (n=1)	MG (n=15 FR e n= 2 C)	Yes	Silveira et al. (2011)
			Blood smear and nPCR (18S rRNA)	439 to 820	Liver, brain, and lymph node	4/8 (50%)– blood smear 5/9 (55,6%)– nPCR	*Theileria* sp. phylogenetically related to *T. cervi*	MG (FR)	No	Silveira et al. (2014)
			Blood smear and cPCR	561	Blood	1/6 (16.7%) – Blood smear and 1/6 - PCR	*Theileria cervi*	GO and/or MG (C)	No	Fava et al. (2022)
			nPCR (18S rRNA), cPCR (18S rRNA, *hsp70*)	1590 – 18S rRNA 599 to 620 – *hsp70*	Buffy coat	22/26 (84.6%)	*Theileria* sp. (“*Theileria* sensu stricto” clade)	GO (n=1), MS (n=19), SP (n=2) (FR)	-	Calchi et al. (2024a)
			cPCR (18S rRNA, *cox-3, hsp70* and ITS-1)	~1350 – 18S rRNA 650 – *cox-3* 810 – *hsp70* 534 – ITS-1	Blood	9/12 (75%)	*Theileria* sp. (“*Theileria* sensu stricto” clade)	GO (FR)	No	Bittencourt et al. (2025)
	Hippopotamidae	*Hippopotamus amphibius* (common hippopotamus)^*^	nPCR (18S rRNA)	430	Blood	1/1	No sequence	MG (C)	No	Castillo et al. (2024)
	Tayassuidae	*Pecari tacaju* (collared peccary)	Blood smear	-	Blood	1/1 – Blood smear	-	GO and/or MG (C)	No	Fava et al. (2022)
		*Tayassu pecari* (White-lipped Peccary)	cPCR (18S rRNA)	514	Lung and liver	1/9 (11.1%)	*Babesia* sp. phylogenetically related to *Babesia* sp. WA1	PA (FR)	**-**	Soares et al. (2017)
Carnivora	Canidae	*Cerdocyon thous* (crab-eating fox)	cPCR (18S rRNA)	549	Blood, bone maarrow and/or tissues	6/20 (30%)	*R. vitalii*	RS (n=5) and SP (n=1) (FR)	No	Soares et al. (2014)
			cPCR (18S rRNA)	500	Liver	1 (Case report)	*R. vitalii*	RS (FR)	Yes	Fredo et al. (2015)
			nPCR (18S rRNA)	739	Blood	1/78 (1.3%)	*Babesia* sp. phylogenetically related to *B. caballi*	MS (FR)	No	Sousa et al. (2018)
			Blood smear and nPCR (18S rRNA)	593	Blood	1 (Case report)	*R. vitalii*	RS (FR)	Yes	Copat et al. (2019)
			cPCR (18S rRNA)	500	Blood	7/27 (25.9%)	*R. vitalii*	RS (FR)	No	Souza et al. (2019)
			qPCR (*hsp70*)	180	Tissues	8/31 (25.8%)	*R. vitalii*	RS (FR)	No	de Lorenzo et al. (2021)
			Blood smear and cPCR	1684	Blood	5/10 (50%)- blood smear 4/10 (40%)- PCR	No sequence	GO and/or MG (C)	No	Fava et al. (2022)
			qPCR (*hsp70*) and cPCR (18S rRNA)	495	Blood	2/3 (66.7%)	*R. vitalii*	SC (FR)	No	Souza et al. (2023)
			cPCR (18S rRNA, *hsp70*) and nPCR (*cox-1*)	1350 to 1470 – 18S rRNA 980 – *hsp70* ~770 – *cox-1*	Blood	3/12 (25%)	*B. pantanalensis* (*Babesia* sensu stricto clade)	MS (FR)	No	Calchi et al. (2024b)
			cPCR (18S rRNA)	551	Blood	2/11 (18.2%)	No sequence	GO (FR)	No	Bittencourt et al. (2025)
		*Chrysocyon brachyurus* (Maned wolf)	Blood smear	-	Blood	1 (Case report)	*B. canis*	DF (FR)	Yes	Cansi et al. (2012)
			cPCR (*hsp70*)	614	Mesenteric lymph node	1 (Case report)	*R. vitalii*	MG (FR)	Yes	Silveira et al. (2016)
			cPCR	1684	Blood	1/6 (16.7%)	No sequence	GO and/or MG (C)	No	Fava et al. (2022)
			cPCR (18S rRNA)	551	Blood	2/6 (33.4%)	No sequence	GO (FR)	No	Bittencourt et al. (2025)
		*Lycalopex gymnocercus* (Pampas fox)	cPCR (18S rRNA)	492	Blood	1 (Case report)	*R. vitalii*	SC (FR)	Yes	de Quadros et al. (2015)
			cPCR (18S rRNA)	500	Liver	1 (Case report)	*R. vitalii*	RS (FR)	Yes	Fredo et al. (2015)
			Blood smear and nPCR (18S rRNA)	1307	Blood	1 (Case report)	*R. vitalii*	SC (FR)	No	Silva et al. (2018)
			cPCR (18S rRNA)	500	Blood	1/17 (5.9%)	*R. vitalii*	RS (FR)	No	Souza et al. (2019)
		*Lycalopex vetulus* (Hoary fox)	Blood smear and cPCR	1684	Blood	1/5 (20%)- Blood smear 3/5 (60%) – PCR	No sequence	GO and/or MG (C)	No	Fava et al. (2022)
	Felidae	*Leopardus braccatus* (Pantanal cat)	nPCR (18S rRNA)	790	Blood	1/5 (20%)	*Babesia* sp. (97 to 99% identity with B. leo)	SP (C)	No	André et al. (2011)
			nPCR (18S rRNA)	480	Blood	2/2	No sequence	MG (C)	No	Castillo et al. (2024)
		*Leopardus pardalis* (ocelot)	cPCR (18S rRNA)	~400	Blood	6/29 (20.7%)	*Cytauxzoon* sp.	DF (n=2) and SP (n=4) (C)	No	André et al. (2009)
			cPCR (18S rRNA)	1598	Blood	1/16 (6.2%)	*C. brasiliensis*	SP (C)	No	Filoni et al. (2012)
			cPCR (18S rRNA)	508	Lung and liver	1/1	*Cytauxzoon* sp.	PA (FR)	**-**	Soares et al. (2017)
			Blood smear nPCR (18S rRNA)	~250 or ~750	Blood	1/7 (14,3%) – blood smear 3/7 (42,9%) – PCR	*Cytauxzoon* sp.	MS (FR)	No	Sousa et al. (2018)
			nPCR (18S rRNA)	721	Spleen	1/4 (25%)	*Cytauxzoon* sp.	MT (FR)	No	Silva et al. (2021)
			cPCR (18S rRNA)	284	Blood	4/5 (80%)	*Cytauxzoon* sp.	MT and MS (VL)	No	Fagundes-Moreira et al. (2022)
			Blood smear and cPCR	574	Blood	1/3 (33,3%)– blood smear 3/3 – PCR	*Cytauxzoon* sp.	GO and/or MG (C)	No	Fava et al. (2022)
			nPCR (18S rRNA e *cox-1*), cPCR (18S rRNA, *cytB*, ITS-1 and ITS-2)	~1550 – 18S rRNA ~1089 – *cytB* 1254 – *cox1* 450 - ITS1 237 - ITS2	Blood	10/13 (76.9%)	*C. brasiliensis*	MS (FR)	-	Calchi et al. (2025b)
			cPCR (18S rRNA and *cytB*)	900 to 1640 – 18S rRNA 1027 to 1084 - *cytB*	Blood	21/27 (73.52%)	*C. brasiliensis* and *C. felis*	MG and MT (FR)	No	May et al. (2025)
		*Leopardus tigrinus* (oncilla)	cPC (18S rRNA)	1202	Blood	1 (Case report)	*Cytauxzoon* sp.	RJ (C)	-	Amaral (2006)
			Blood smear and nPCR (18S rRNA, *cytB*)	1459 – 18S rRNA 1097 - *cytB*	Blood	1 (Case report)	*C. brasiliensis*	DF (FR)	No	Duarte et al. (2024)
		*Panthera leo* (lion)*	Clinical signs, necropsy, and histopathology	-	Tissues	2 (Case report)	*Cytauxzoon* sp.	RJ (C)	Yes	Peixoto et al. (2007)
		*Panthera onca* (jaguar)	cPC (18S rRNA)	947	Blood	1 (Case report)	*Cytauxzoon* sp.	RJ (C)	-	Amaral (2006)
			cPCR (18S rRNA)	~400	Blood	1/9 (11.1%)	*Cytauxzoon* sp.	DF (C)	No	André et al. (2009)
			cPCR (18S rRNA)	330	Blood	28/29 (96.5%)	*Cytauxzoon* sp.	GO (n=4), MS (n=22), TO (n=3) (FR)	No	Furtado et al. (2017)
			cPCR (18S rRNA – ITS1)	525	Tissues	1 (Case report)	*Cytauxzoon* sp.	MS (C)	Yes	Guizelini et al. (2021)
			nPCR (18S rRNA)	721	Spleen	3/6 (50%)	*Cytauxzoon* sp.	MT (C)	-	Silva et al. (2021)
			cPCR (18S rRNA)	284	Blood	45/45	*Cytauxzoon* sp.	MT e MS (VL)	No	Fagundes-Moreira et al. (2022)
			nPCR (18S rRNA)	430	Blood	1/1	*Cytauxzoon* sp.	MG (C)	No	Castillo et al. (2024)
			qPCR (18S rRNA)	~220	Blood	2/2	*Cytauxzoon* sp.	MS (FR – roadkill)	-	Alves et al. (2025)
			nPCR (18S rRNA e *cox-1*), cPCR (18S rRNA and *cytB*)	1380 to 1466 – 18S rRNA 1044 to 1131 – *cytB* ~1250 – *cox-1*	Blood	41/41	*C. felis*	AM (n=1), BA (n=2), ES (n=1), GO (n=17), MG (n=1), MS (n=9), MT (n=3), PA (n=2), PI (n=1), PR (n=2), TO (n=1)	-	Calchi et al. (2025b)
		*Puma concolor* (puma)	Blood smear	-	Blood	2/7 (28.6%)	*Cytauxzoon* sp.	MS (FR and C)	No	Juliano et al. (2004)
			cPCR (18S rRNA)	~400	Blood	2/9 (22.2%)	*Cytauxzoon* sp.	DF (C)	No	André et al. (2009)
			Blood smear	-	Blood	1 (Case report)	*Cytauxzoon* sp.	MS (FR)	No	Antunes et al. (2018)
			Blood smear and cPCR (18S rRNA)	600	Blood	6/11 (54.5%)	*Cytauxzoon* sp.	MS (C)	No	Silva et al. (2020)
			nPCR (18S rRNA)	721	Blood	2/8 (25%)	*Cytauxzoon* sp.	MT (FR)	-	Silva et al. (2021)
			Blood smear and cPCR (18S rRNA)	455	Blood	1 (Case report)	*Cytauxzoon* sp.	GO (FR)	No	Paula et al. (2022)
			cPCR (18S rRNA)	284	Blood	3/3	*Cytauxzoon* sp.	MT and MS (FR)	No	Fagundes-Moreira et al. (2022)
			Blood smear	-	Blood	2/12 (16.7%)	-	GO and/or MG (C)	No	Fava et al. (2022)
			Blood smear and cPCR (18S rRNA)	1270	Blood and lung	1/6 blood smear 4/6 PCR	*C. brasiliensis*	GO (FR)	No	Bittencourt et al. (2025)
			cPCR (18S rRNA and *cytB*)	850 and 1638 – 18S rRNA 1039 - *cytB*	Blood	6/9 (60%)	*C. brasiliensis* and *C. felis*	PA and MS (FR)	No	May et al. (2025)
	Viverridae	*Genetta genetta* (common genet)*	nPCR (18S rRNA)	526	Blood	1/2 (50%)	*Babesia* sp. (97 to 99% identity with *B. leo*)	SP (C)	No	André et al. (2011)
	Mustelidae	*Eira arbara* (tayra)	cPCR (18S rRNA)	1684	Blood	1/1	No sequence	GO and/or MG (C)	No	Fava et al. (2022)
	Procyonidae	*Nasua nasua* (coati)	Blood smear and nPCR (18S rRNA)	450 a 564	Blood	1/31 (3.2%) – blood smear 3/31 (9.7%) – PCR	*Theileria* sp. phylogenetically related to *Theileria* sp. detected in a cat from Brazil	MS (FR)	No	Sousa et al. (2018)
			nPCR (18S rRNA)	~380	Blood	3/209 (1.4%)	*Babesia* sp. phylogenetically related to *B. bigemina*	MG (FR)	No	Estevam et al. (2020)
			cPCR (18S rRNA)	1684	Blood	2/2	No sequence	GO and/or MG (C)	No	Fava et al. (2022)
		*Procyon cancrivorus* (crab-eating Raccoon)	Blood smear and cPCR	1684	Blood	1/2 (50%)– blood smear 2/2 – PCR	No sequence	GO and/or MG (C)	No	Fava et al. (2022)
Chiroptera	Phyllostomidae	*Artibeus lituratus* (Great Fruit-eating Bat)	nPCR and cPCR (18S rRNA)	708	Blood	1/37 (2.7%)	*Piroplasmid* sp. (“*Babesia* sensu stricto” clade)	MS (FR)	-	Ikeda et al. (2021)
		*Artibeus planirostris* (flat-faced fruit-eating Bat)	nPCR and cPCR (18S rRNA)	741	Blood and spleen	4/33 (12.1%)	*Piroplasmid* sp. (“*Babesia* sensu stricto” clade)	MS (FR)	-	Ikeda et al. (2021)
		*Desmodus rotundus* (common vampire bat)	nPCR and cPCR (18S rRNA)	~1500	Spleen	42/228 (18.4%)	*Babesia* sp. (“South American Marsupialia Group”) *Theileria* sp. (“Theileria sensu stricto” clade and “Tapirus terrestres group”)	AP (n=1), PA (n=37), RR (n=4) (FR)	-	Mello et al. (2023a)
		*Diaemus youngi* (white-winged vampire bat)	nPCR and cPCR (18S rRNA)	-	Spleen	1/1	No sequence	PA (FR)	-	Mello et al. (2023a)
		*Platyrrhinus lineatus* (white-lined broad-nosed bat)	nPCR and cPCR (18S rRNA)	-	Spleen	2/23 (8.7%)	No sequence	MS (FR)	-	Ikeda et al. (2021)
		*Phyllostomus discolor* (Pale spear-nosed bat)	nPCR and cPCR (18S rRNA)	1455 to 1570	Blood and spleen	10/15 (66.7%)	*Piroplasmid* sp. (“Phyllostomatidae bat Group” clade)	MS (FR)	-	Ikeda et al. (2021)
Cingulata	Chlamyphoridae	*Euphractus sexcinctus* (six-banded armadillo)	nPCR (18S rRNA)	-	Blood or spleen	1/11 (9.1%)	No sequence	MS (FR)	-	Calchi et al. (2023)
		*Priodontes maximus* (giant armadillo)	nPCR (18S rRNA)	638 to 728	Blood	4/32 (12.5%)	*Babesia* sp. (“South American Marsupialia clade)	MS (FR)	-	Calchi et al. (2023)
	Dasypodidae	*Dasypus novemcinctus* (nine-banded armadillo)	cPCR (18S rRNA)	473	Lung and liver	3/32 (9.4%)	*Theileria* sp. phylogenetically related to *Theileria* sp. detected in agouti”	MT (n=1) and PA (n=2) (FR)	**-**	Soares et al. (2017)
			nPCR (18S rRNA),	731	Blood or spleen	3/10 (30%)	*Theileria* sp. phylogenetically related to *Theileria* sp. detected in the same host	MS (n=1) and SP (n=2) (FR)	-	Calchi et al. (2023)
Didelphimorphia	Didelphidae	*Didelphis albiventris* (white-eared opossum)	Blood smear, nPCR and cPCR (18S rRNA), nPCR (*cox-1*), cPCR (*hsp70*)	1463 – 18S rRNA 434 to 575 – *hsp70* 665 and 708 – *cox-1*	Blood	10/67 (14.9%) – blood smear 22/67 (32.8%) - PCR	*Babesia* sp. (“South American Marsupialia Group”)	DF (n=24) MS (n=43) (FR)	-	Gonçalves et al. (2021)
			Blood smear and cPCR (18S rRNA)	1684	Blood	5/26 (19.2%)– blood smear 4/26 (15.4%)- PCR	No sequence	GO and/or MG (C)	No	Fava et al. (2022)
		*Didelphis aurita* (black-eared opossum)	Blood smear, nPCR and cPCR (18S rRNA) and cPCR (*cox-3*)	1569 and 1609 – 18S rRNA 771 and 776 – *cox-3*	Blood and bone marrow	2/15 (13.3%)– Blood smear 5/15 (33.3%)- PCR	*Babesia* sp. (“South American Marsupialia Group)	RJ (FR)	Yes (1 animal)	Oliveira et al. (2023)
		*Didelphis marsupialis* (common opossum)	cPCR (18S rRNA)	524	Lung and liver	1/17 (5.9%)	*Babesia* sp. phylogenetically related to *Babesia* sp. detected in short-tailed opossum	PA (FR)	**-**	Soares et al. (2017)
			cPCR (18S rRNA)	525	Blood	2/31 (6.5%)	*Babesia* sp. phylogenetically related to *Babesia* sp. detected in opossum	MT (FR)	**-**	Colle et al., 2019
			nPCR (18S rRNA)	769	Blood	1/45 (2.2%)	*Babesia* sp. (“South American Marsupialia Group)	MA (FR)	-	Braga et al. (2023)
		*Monodelphis domestica* (gray short-tailed opossum)	cPCR (18S rRNA)	556	Blood, spleen and liver	1/2 (50%)	*Babesia* sp. phylogenetically related to *Babesia* sp. detected in *Thrichomys* sp.	MT (FR)	-	Wolf et al. (2016)
Perissodactyla	Rhinoceratidae	*Ceratotherium simum* (white rhinoceros)*	nPCR (18S rRNA)	430	Blood	1/2 (50%)	No sequence	MG (C)	No	Castillo et al. (2024)
	Tapiridae	*Tapirus terrestris* (tapir)	cPCR (18S rRNA)	414	Blood	1 (Case report)	*Theileria* sp. 98% identical to *T. equi*	MS (FR)	No	Silveira et al. (2017)
			Blood smear and cPCR (18S rRNA)	392 to 475	Blood	7/19 (36.8%) blood smear 11/19 (57.9%) – PCR	*T. equi*	AM (n=1) and PA (n=18) (C)	No	Gonçalves et al. (2020)
			nPCR (18S rRNA)	776	Spleen and blood	6/17 (35.3%)	*Theileria* sp. phylogenetically related to *Theileria* sp. detected in cat from Brazil	MT (FR)	-	Silva et al. (2021)
			Blood smear and cPCR	553	Blood	1/2 (50%)– blood smear 1/2 – PCR	*Theileria* sp. phylogenetically related to *T. equi*	GO and/or MG (C)	No	Fava et al. (2022)
			Blood smear, nPCR (18S rRNA and *cox1*), cPCR (*hsp70*)	1200 to 1500 – 18S rRNA 687 to 782 – *hsp70* 354 to 410 – *cox-1*	Blood	56/99 (56.7%)- PCR	*T. terrestris* (“*Tapirus terretris”* group)	MS (FR)	-	Mongruel et al. (2022)
			cPCR (18S rRNA. *hsp70*)	1350 and 1397 – 18S rRNA 739 and 893 – *hsp70*	Blood, liver and spleen	4/4	*T. terrestris* (“*Tapirus terrestris”* group)	GO (FR and C)	No	Bittencourt et al. (2025)
Pilosa	Myrmecophagidae	*Myrmecophaga tridactyla* (giant anteater)	Blood smear and cPCR (18S rRNA)	1684	Blood	6/21 (28.6%) – blood smear 2/21 (9.5%) - PCR	No sequence	GO and/or MG (C)	No	Fava et al. (2022)
			nPCR (18S rRNA), cPCR (ITS-1)	-	Blood or spleen	12/131 (9.2%)	No sequence	MS (n=7) and SP (n=4) (FR)	-	Calchi et al. (2023)
			cPCR (18S rRNA)	551	Blood	2/21 (9.5%)	No sequence	GO (FR)	No	Bittencourt et al. (2025)
		*Tamandua tetradactyla* (southern tamandua)	Blood smear and cPCR (18S rRNA)	1684	Blood	5/11(45.5%) – blood smear 2/11 (18.2%) - PCR	No sequence	GO and/or MG (C)	No	Fava et al. (2022)
			nPCR (18S rRNA), cPCR (ITS-1)	-	Blood or spleen	5/37 (13.5%)	No sequence	MS (n=1) and SP (n=4) (FR)	-	Calchi et al. (2023)
Primates	Atelidae	*Alouatta caraya* (black-and-gold howler monkey)	Blood smear and cPCR (18S rRNA)	1684	Blood	2/5 (40%) – blood smear 1/5 (20%) – PCR	No sequence	GO and/or MG (C)	No	Fava et al. (2022)
		*Ateles* spp. (spider monkeys)	nPCR (18S rRNA)	430	Blood	1/1	No sequence	MG (C)	No	Castillo et al. (2024)
	Cebidae	*Callithrix penicillata* (black-tufted-ear Marmoset)*	Blood smear and cPCR	1684	Blood	11/23 (47.8%)– blood smear 4/23 (17.4%)– PCR	No sequence	GO and/or MG (C)	No	Fava et al. (2022)
		*Sapajus apella* (tufted capuchin)	nPCR (18S rRNA)	430	Blood	1/2 (50%)	No sequence	MG (C)	No	Castillo et al. (2024)
	Hominidae	*Gorilla gorila* (gorilla)*	nPCR (18S rRNA)	430	Blood	2/3 (66.7%)	No sequence	MG (C)	No	Castillo et al. (2024)
Rodentia	Caviidae	*Hydrochoerus hydrochaeris* (capybara)	cPCR (18S rRNA)	1714	Blood	2/14 (14.3%)	*Babesia* sp. Capybara	RS (FR)	-	Criado-Fornelio et al. (2009)
			cPCR (18S rRNA)	1684	Blood	1/2 (50%)	No sequence	GO and/or MG (C)	No	Fava et al. (2022)
			Blood smear, cPCR (18S rRNA, *hsp70*, *cox-3*)	1519 to 1561 -18S rRNA 825 to 887 –*hsp70* 590 – *cox-3*	Blood	1/28 (3.6%)– blood smear 11/28 (39.3%)- PCR	*B. goianiaensis* (“Capybara group)	GO (FR)	-	Krawczak et al. (2023); Neves et al., (2023)
			cPCR (18S rRNA)	565	Blood,	2/4 (50%)	*B. goianiaensis* (100% identity)	GO (FR)	No	Bittencourt et al. (2025)
	Cuniculidae	*Cuniculus paca* (lowland paca)	cPCR (18S rRNA)	537	Lung and liver	4/33 (12.1%)	*Babesia* sp. phylogenetically related to *Babesia* sp. detected in capybara	MT (FR)	**-**	Soares et al. (2017)
	Dasyproctidae	*Dasyprocta* sp. (agouti)	cPCR (18S rRNA)	314	Lung and liver	1/1	*Theileria* sp. phylogenetically related to *Theileria* sp. detected in armadillo	MT (FR)	**-**	Soares et al. (2017)
	Echimyidae	*Thrichomys fosteri* (Foster's Punaré)	cPCR (18S rRNA)	~720	Blood	6/77 (7.8%)	*Babesia* sp. phylogenetically related to *Babesia vogeli* and *Theileria* sp. related to *T. equi*	MS (FR)	No	Sousa et al. (2018)
		*Thrichomys pachyurus* (Paraguayan Punaré)	cPCR (18S rRNA)	532	Blood, spleen and liver	3/11 (27.3%)	*Babesia* sp. phylogenetically related to *Babesia* sp. detected in *Monodelphis*	MT (FR)	-	Wolf et al. (2016)
			cPCR (18S rRNA and *cytB)* nPCR (18S rRNA)	1200 to 1230 – 18S rRNA 722 to 830 - *cytB*	Blood, lung and spleen	17/93 (18.3%)	*Piroplasmida* sp. (“South American Rodentia Group”)	MT (FR)	-	Pacheco et al. (2025)
	Erethizontidae	*Coendou prehensilis* (Brazilian porcupine)	cPCR (18S rRNA)	1684	Blood	7/10 (70%)– PCR	No sequence	GO and/or MG (C)	No	Fava et al. (2022)
			cPCR (18S rRNA)	304	Blood	1/6 (16.7%)	*B. vogeli* (100% identity)	GO (FR)	No	Bittencourt et al. (2025)
		*Sphiggurus spinosus* (Paraguayan hairy dwarf porcupine)	Blood smear	-	Blood	1/1	-	GO and/or MG (C)	No	Fava et al. (2022)
**Birds**										
Anseriformes	Anatidae	*Neochen jubata* (Orinoco goose)	nPCR (18S rRNA)	693	Blood	6/62 (9.7%)	*Babesia* sp. phylogenetically related to *B. vogeli*	GO (FR)	-	Werther et al. (2017)
Procellariiformes	Diomedeidae	*Thalassarche chlororhynchos* (Atlantic yellow-nosed albatross)	cPCR (18S rRNA)	672	Blood	1/15 (6,7%)	*Babesia* sp. (*Babesia* sensu stricto clade)	SC (FR)	-	Sgarioni et al. (2023)
Suliformes	Sulidae	*Sula dactylatra* (masked booby)	cPCR (18S rRNA)	-	Blood	22/168 (13.1%)	No sequence	Islands in the Northeast of Brazil (FR)	-	Quillfeldt et al. (2014)
		*Sula leucogaster* (brown booby)	cPCR (18S rRNA)	1450	Blood	12/172 (7.0%)	*Babesia* sp. (Peircei clade)	Islands in the Northeast of Brazil (VL)	-	Quillfeldt et al. (2014)

* Exotic animals in Brazil.

## Order Artiodactyla

### Family Cervidae

In Brazil, most of the genotypes (fragments of 400 to 500 bp) of the 18S rRNA gene of piroplasmids detected in cervids correspond to *Theileria cervi* and have been described in gray brocket deer (*Subulo gouazoubira*) (52.9% [9/17]) and marsh deer (*Blastocerus dichotomus*) (25% [1/4]) sampled in the state of Minas Gerais (Silveira et al., 2011), Pampas deer (*Ozotocerus bezoarticus*) (20% [12/60]) sampled in the Pantanal of the state of Mato Grosso do Sul (Silveira et al., 2013), and in red brocket deer (*Mazama americana*) (100% [2/2]) in the state of Pará (Soares et al., 2017).

*Babesia bovis* was detected by PCR in marsh deer (1/4) and Pampas deer (3/60) sampled in the states of Minas Gerais and Mato Grosso do Sul, respectively. *Babesia bigemina* was detected in gray brocket deer (1/12) and Pampas deer (2/60) sampled in the aforementioned states (Silveira et al., 2011; Silveira et al., 2013). All abomentioned studies used blood samples for Piroplasmida detection. Additionally, antibodies against *B. bovis* and *B. bigemina* were detected in marsh deer sampled near the Porto Primavera Hydroelectric Power Station, located on the Paraná River in the state of Paraná, Brazil (Machado et al., 2001).

In a recent study, a high prevalence of piroplasmids was detected in buffy coat samples from Neotropical deer using nested PCR (nPCR): 73.2% (101/138) in marsh deer, 100% (4/4) in small red brocket deer (*Mazama jucunda*), 100% (3/3) in southern red brocket deer (*Mazama rufa*), 60% (6/10) in pampas deer, and 84.6% (22/26) in gray brocket deer sampled in different states of the country. The sequences obtained from the *hsp70*, *cox-3*, and 18S rRNA near-complete genes were positioned within the *Theileria* sensu stricto clade, close to *Theileria cervi* and *Theileria capreoli* (Calchi et al., 2024a).

Also, Bittencourt et al. (2025) reported positivity rates of 75% (9/12) in gray brocket deer and 100% (1/1) in red brocket deer sampled in the state of Goiás, based on PCR analysis of DNA extracted from blood samples. Phylogenetic analyses based on near-complete 18S rRNA, *hsp70*, and *cox-3* genes placed the sequences obtained in gray brocket deer within the “*Theileria* sensu stricto” clade, closely related to *Theileria* sp. sequences previously detected in deer (including the same host species) by Calchi et al. (2024a), as well as to *T. cervi*. ITS-1 sequences of *Theileria* sp. from *S. gouazoubira* were also placed within the “*Theileria* sensu stricto” clade.

### Family Tayassuidae

One peccary – *Tayassu pecari* (11.1% [1/9]) sampled in the state of Pará was PCR positive for piroplasmids. The 514 bp sequence of the 18S rRNA gene was positioned in a clade close to a *Babesia* sp. WA1 sequence detected in a child from California and to a clade encompassing *Babesia lengau*, *B. gibsoni*, and *Babesia conradae* (Soares et al., 2017).

## Order Carnivora

### Family Canidae

The molecular prevalence for piroplasmids reported in wild canids ranged from 1.3% (1/78) to 66.6% (2/3) (Soares et al., 2014; Sousa et al., 2018; Souza et al., 2019; Lorenzo et al., 2021; Fava et al., 2022; Souza et al., 2023; Calchi et al., 2024b; Bittencourt et al., 2025). The species described as circulating in these animals in the country were *Rangelia vitalii, Babesia* sp. phylogenetically related to *Babesia caballi*, and a novel *Babesia* species (*Babesia pantanalensis*) (Soares et al., 2014; Sousa et al., 2018; Calchi et al., 2024a). In addition, Cansi et al. (2012) reported a clinical case of a maned wolf (*Chrysocyon brachyurus*) presenting with puncture wounds with myiasis, prostration, normochromic normocytic anemia, and anisocytosis at the Brasília Zoo. Inclusions in erythrocytes were observed and morphologically diagnosed as *B. canis*. The animal died while under treatment. Necropsy revealed hepatomegaly and splenomegaly, ecchymoses on the penile body, and destruction of the medullary and cortical layers of the kidneys due to parasitism by *Dioctophyme renale*. Since no additional analyses were performed to confirm the species of the piroplasmid found, it was not possible to definitively say that it was *B. canis*, as this species is morphologically similar to other *Babesia* spp. and *R. vitalii* species, and it has never previously been diagnosed by molecular techniques in the country.

*Rangelia vitalii* has been reported in wild canids of the species *Cerdocyon thous* (crab-eating fox) (Soares et al., 2014; Fredo et al., 2015; Copat et al., 2019; Souza et al., 2019; Lorenzo et al., 2021; Souza et al., 2023), *Chrysocyon brachyurus* (Silveira et al., 2016), and *Lycalopex gymnocercus* (Pampas fox) (Quadros et al., 2015; Fredo et al., 2015; Silva et al., 2018; Souza et al., 2019). The parasite occurs in the south and southeast of the country and is associated with the presence of the tick vector *Amblyomma aureolatum* (Soares et al., 2018). *Cerdocyon thous* is considered the natural host and reservoir of this agent, as it is the animal species most frequently reported in the literature, because it typically does not develop clinical signs (the schizogony phase is suspected to be brief and self-limiting), it is the primary host for *A. aureolatum* and can maintain the infection (Labruna et al., 2005; Soares et al., 2014; Lorenzo et al., 2021; Souza et al., 2023). Soares et al. (2014) monitored a *C. thous* individual for 80 days, during which it remained PCR positive for *R. vitalii*; the animal eventually died from unknown causes. Additionally, Souza et al. (2023) captured a specimen of *Cerdocyon thous* that showed to be PCR positive for *R. vitalii* at the first sampling and 93 days later. Interestingly, at the time of the first capture, the animal showed low platelet, erythrocyte, hematocrit, and hemoglobin counts. Upon recapture, these values had slightly increased, which might indicate that the animal was in the acute phase of the infection during the first capture and remained chronically infected thereafter (Souza et al., 2023).

Quadros et al. (2015) described the case of a free-living Pampas fox female in Santa Catarina who presented pale mucous membranes, apathy, hypothermia, dehydration, and motor incoordination. Laboratory tests showed the presence of lymphocytosis, neutropenia, polychromasia, erythroblastosis, and Howell-Jolly corpuscles. The animal died, and infection by *R. vitalii* was confirmed by PCR based on the 18S rRNA gene and by the presence of structures morphologically compatible with this hemoparasite in kidney and heart tissues. In addition to *R. vitalii*, the animal was co-infected with *Hepatozoon canis* and *Calodium hepaticum*.

In 2019, Copat et al. reported a clinical case of a *Cerdocyon thous* rescued in Rio Grande do Sul, presenting with jaundice, motor incoordination, blackish feces, and infestation by *Amblyomma aureolatum*. Pyriform inclusions in red blood cells were detected in the blood smear; the blood count showed normochromic normocytic anemia, anisocytosis, polychromasia, leukocytosis, neutrophilia, monocytosis, and thrombocytopenia. Biochemical tests showed increased levels of urea and creatinine, hypoalbuminemia, and hypoproteinemia. At necropsy, generalized jaundice, splenomegaly, bleeding in the pancreas, petechiae on the intestinal mucosa, and melena were found. Histological sections showed the presence of the *R. vitalii* in the vascular endothelium of various organs. Parasite DNA was detected by PCR (Copat et al., 2019).

Other studies have also reported the presence of this parasite in the cytoplasm of endothelial cells of different organs in a maned wolf in Minas Gerais (Silveira et al., 2016) and a Pampas fox and a crab-eating fox in Rio Grande do Sul (Fredo et al., 2015). In both studies, the animals showed various non-specific clinical signs and died, but it was not possible to determine whether the clinical signs and cause of death were due to *R. vitalii* infection, as the animals had co-infection with other agents that could cause the same clinical signs. The maned wolf was parasitized by various helminths species and protozoan parasites, as well as *Hepatozoon* sp., *Leishmania* sp., and *Entamoeba* sp.; the Pampas fox had been attacked by a dog; and the crab-eating fox was infected with the distemper virus (Fredo et al., 2015; Silveira et al., 2016). *Cerdocyon thous* infected with *R. vitalii* presented fewer schizonts, particularly in non-hematopoietic organs, compared to domestic dogs. This may be related to the absence of clinical signs in these animals, except in rare cases (Lorenzo et al., 2021).

A *Babesia* sp. phylogenetically related to *B. caballi* was detected by Sousa et al. (2018) in a *Cerdocyon thous* (1.2% [1/78]- blood samples) sampled in the southern Pantanal region of Mato Grosso do Sul. Unfortunately, the fragment obtained from the 18S rRNA gene was relatively short (739 bp), which limited a more accurate interpretation of its phylogenetic position.

A new species of *Babesia*, named *Babesia pantanalensis* nov. sp., was described in crab-eating fox sampled in the Pantanal region of Mato Grosso do Sul (Calchi et al., 2024b). The description of this new species was based on phylogenetic analyses using multiple molecular markers (near-complete fragment of the 18S rRNA gene, *hsp70*, and *cox1*), which placed the obtained sequences in a clade within the *Babesia* sensu stricto group, as a sister clade to *Rangelia vitalii*. The genetic divergence between the species ranged from 4.17% to 5.62% for the 18S rRNA gene, 6.16% for *hsp70*, and 4.91% to 9.25% for *cox1* (Calchi et al., 2024b). Furthermore, the absence of *Amblyomma aureolatum* in the central-western region of the country reinforces the classification of *B. pantanalensis* as a distinct species from *R. vitalii*, since this is typically found in high-altitude areas of the Atlantic Forest and Pampa biomes (Labruna et al., 2005, 2025). *Amblyomma parvum* is a putative vector of *B. pantanalensis*, as *hsp70* sequences closely with *R. vitalii* were detected in nymphs of this tick species collected from wild rodents in the Pantanal region of Mato Grosso do Sul (Sousa et al., 2018).

### Family Felidae

To date, the genus of piroplasmids most frequently reported in wild felids in Brazil is *Cytauxzoon* spp. This agent has been detected in both captive and free-living individuals of the following species: *Leopardus pardalis* (ocelot), *Leopardus tigrinus* (little spotted cat), *Leopardus braccatus* (Pantanal cat), *Panthera onca* (jaguar), and *Puma concolor* (puma) (Juliano et al., 2004; Amaral, 2006; André et al., 2009; Filoni et al., 2012; Furtado et al., 2017; Soares et al., 2017; Antunes et al., 2018; Sousa et al., 2018; Silva et al., 2020; Guizelini et al., 2021; Silva et al., 2021; Paula et al., 2022; Fagundes-Moreira et al., 2022; Fava et al., 2022; Castillo et al., 2024; Duarte et al., 2024; Alves et al., 2025; Calchi et al., 2025b; Bittencourt et al., 2025; May et al., 2025).

Molecular prevalence ranged from 0.60% (1/167) to 98.1% (52/53) in the studies conducted (André et al., 2009; Filoni et al., 2012; Furtado et al., 2017; Sousa et al., 2018; Silva et al., 2020; Silva et al., 2021; Fagundes-Moreira et al., 2022; Alves et al., 2025; Calchi et al., 2025b; Bittencourt et al., 2025; May et al., 2025).

Although most studies conducted in Brazil report the occurrence of *Cytauxzoon felis* in wild felids, these findings were based on a short fragment of the 18S rRNA gene, which is highly conserved and limits accurate species identification. Duarte et al. (2024), using a nearly complete fragment of the 18S rRNA gene (1,459 bp) and a large fragment of the *cytB* gene (1,097 bp), described a new species in the country, named *Cytauxzoon brasiliensis*, first detected in *Leopardus tigrinus*. This species was placed in a distinct clade, though closely related to the *C. felis* clade. The genetic divergence between *C. felis* and *C. brasiliensis* was 0.4% for the 18S rRNA gene and approximately 6.8% for the *cytB* gene.

Recently, the occurrence of at least two species of *Cytauxzoon* spp. was confirmed through phylogenetic inferences based on multiple molecular markers (nearly complete 18S rRNA, *cox1*, *cytB*, and the intergenic regions ITS1 and ITS2) in wild felids sampled in the country. *Cytauxzoon brasiliensis* was detected in ocelots sampled in the Pantanal region of Mato Grosso do Sul, while genovariants of *C. felis* were identified in jaguars sampled in various states of Brazil and in Argentina (Calchi et al., 2025b). Similar findings were reported by May et al. (2025), who identified *C. felis* and *C. brasiliensis* circulating in wild felids in Brazil, based on the amplification of near-complete 18S rRNA and *cytB* genes. In that study, both *Cytauxzoon* species were detected in ocelots and pumas sampled across the Pantanal, Cerrado, and Amazon biomes. Additionally, *C. brasiliensis* was also detected in a puma from state of Goiás, based on amplification of a 1270 bp fragment of the 18S rRNA gene (Bittencourt et al., 2025). These findings reveal a greater diversity of *Cytauxzoon* species circulating in wild felids in Brazil than previously recognized (Calchi et al., 2025b).

A fatal case of cytauxzoonosis was reported in a 5-month-old jaguar cub (*Panthera onca*) born in captivity in Mato Grosso do Sul (Guizelini et al., 2021). The animal presented hyporexia and died despite antibiotic therapy. Necropsy revealed jaundice in the subcutaneous tissue, oral and ocular mucosa, splenomegaly, and a lobular liver. Histopathological examination showed the presence of macrophages containing schizonts obstructing blood vessels in the brain, spinal cord, leptomeninges, lungs, heart, skeletal muscles, adrenal glands, kidneys, spleen, small intestine, and pancreas. The diagnosis was confirmed by PCR and sequencing (Guizelini et al., 2021). Fatal cases of cytauxzoonosis are rare in wild felids. Deaths caused by *Cytauxzoon* spp. infection have been reported in tigers (*Panthera tigris*) kept in captivity in Germany (Jakob & Wesemeier, 1996) and the United States (Garner et al., 1996), as well as in a free-ranging bobcat (*Lynx rufus*) in the United States (Nietfeld & Pollock, 2002).

In the United States, *Lynx rufus* (bobcats) are considered the primary natural hosts and reservoirs of *C. felis*. In these animals, the schizogony phase is short and self-limiting (Glenn et al., 1983). In Brazil, jaguars are suspected to play a similar role in the transmission of *Cytauxzoon* species circulating in the country, due to the high infection rates observed in this host. Several studies have reported *Cytauxzoon* spp. occurrence rates exceeding 96.5% (28/29) in jaguars (Furtado et al., 2017; Fagundes-Moreira et al., 2022; Alves et al., 2025; Calchi et al., 2025b). Furthermore, there are records of individuals recaptured at different times (with intervals between captures ranging from 60 days to up to four years) who remained positive for the parasite. This suggests that these animals either maintain chronic infections or are frequently reinfected (Furtado et al., 2017; Fagundes-Moreira et al., 2022).

Other wild felid species, such as *Leopardus pardalis* and *Puma concolor*, may also play a role as reservoirs of *Cytauxzoon* spp. in the country. In a study conducted by May et al. (2025), individuals from these species that tested positive for the parasite showed no clinical alterations. Additionally, *L. pardalis* individuals recaptured at intervals of 3 to 9 months remained PCR positive, and one individual that initially tested negative was positive upon recapture. These findings suggest a possible role for these species in the maintenance of the parasite in Brazil (May et al., 2025).

There is still no experimental evidence identifying the tick species involved in the transmission of *Cytauxzoon* spp. in Brazil. However, Fagundes-Moreira et al. (2022) suggested *Amblyomma sculptum* as a potential vector, as it was the tick species most frequently found on wild felids that tested positive for *Cytauxzoon* spp. in the states of Mato Grosso and Mato Grosso do Sul. In contrast, all blood samples from wild felids sampled in the state of Rio Grande do Sul tested negative for the parasite, while the positivity rate among animals sampled in the central-western states reached 98.11%. Notably, *A. sculptum* does not occur in Rio Grande do Sul, reinforcing the hypothesis that this species may play a role in the parasite's transmission cycle in other regions. Additionally, *Cytauxzoon* sp. DNA (18S rRNA gene) was detected in *A. sculptum* nymphs collected from wild boars in the state of São Paulo. The authors hypothesized that the larvae might have acquired the infection during blood feeding on domestic or wild felids and maintained it through the nymphal stage (Santana et al., 2022).

Regarding other genera of piroplasmids detected in wild felids in Brazil, André et al. (2011) detected *Babesia* sp. DNA by amplifying a fragment of the 18S rRNA gene in a *Leopardus braccatus* housed in a zoo in the state of São Paulo. The sequence obtained (790 bp) showed 97–98% identity with *Babesia leo*, previously detected in domestic cats and lions in South Africa. Additionally, 31% (54/169) of the wild felids sampled were seropositive for *Babesia vogeli* using the Indirect Fluorescent Antibody Test Assay (IFAT), including the same Pantanal cat that tested PCR-positive.

### Family Procyonidae

There are few reports of piroplasmids in procyonids from Brazil. To date, *Theileria* sp. has been detected, both morphologically and molecularly, in the blood of three coatis (*Nasua nasua*), representing 9.6% (3/31) of the animals sampled in the Pantanal of Mato Grosso do Sul. The three 18S rRNA sequences obtained (fragments ranging from 450 to 564 bp) were placed in the same clade as *Theileria* sp. previously detected in domestic cats in Brazil, forming a sister clade to *T. equi* (Sousa et al., 2018). In contrast, Estevam et al. (2020) detected *Babesia* sp. (380 bp), phylogenetically related to *B. bigemina*, in three coatis (1.4% [3/209]) sampled in a park in Belo Horizonte, state of Minas Gerais. Additionally, two coatis and two crab-eating racoons (*Procyon cancrivorus*) sampled in rehabilitation centers in Goiás and Minas Gerais were PCR-positive for piroplasmid DNA based on the 18S rRNA gene, although no sequences were obtained (Fava et al., 2022).

Perles et al. (2023) found no coatis positive for piroplasmids in specimens sampled in a park and a residential area in the city of Campo Grande, Mato Grosso do Sul. However, piroplasmid DNA was detected in ectoparasites collected from these animals. One pool of *Amblyomma* sp. larvae and two pools of *Amblyomma dubitatum* nymphs tested positive in nested PCR assays targeting the 18S rRNA gene of piroplasmids, with the sequences being placed in the 'South American Marsupialia Group' clade. Additionally, sequences detected in two pools of *Amblyomma sculptum* nymphs were grouped in a clade containing *Babesia* spp. sequences previously detected in capybaras sampled in Campo Grande (MS) and Pelotas (RS), as well as in *A. dubitatum* specimens collected from *Rattus rattus* in Campo Grande. In a study conducted with ectoparasites collected from *Nasua nasua* in Iguaçu National Park, Paraná, *Amblyomma coelebs* nymphs tested positive for *Theileria* sp. The two sequences obtained (fragments of 458 and 588 bp) were placed together in a clade phylogenetically related to the *T. cervi* clade (Araújo et al., 2023).

## Order Chiroptera

### Family Phyllostomidae

In Brazil, two studies reported molecular prevalence rates for piroplasmids of 18.77% (43/229) in non-hematophagous bats and 12.6% (17/135) in hematophagous bats (Ikeda et al., 2021; Mello et al., 2023a).

Specimens from four species of non-hematophagous bats sampled in the peri-urban region of Campo Grande, Mato Grosso do Sul, tested positive in nPCR assays targeting the 18S rRNA gene of piroplasmids: *Artibeus planirostris*, *Artibeus lituratus*, *Phyllostomus discolor*, and *Platyrrhinus lineatus*. Sequences larger than 1450 bp of this gene were obtained from blood DNA samples of *P. discolor* and were positioned within a new clade in the phylogeny, suggesting the possible identification of a new species. This clade was named the 'Phyllostomidae bat group.' In contrast, only short sequences (up to 740 bp) of piroplasmids were obtained from biological samples of *A. planirostris* and *A. lituratus*, which were placed in the '*Babesia* sensu stricto' clade, alongside *B. vogeli* (Ikeda et al., 2021).

Molecular prevalence rates for piroplasmids of 18.4% (42/228) and 100% (1/1) were reported in specimens of *Desmodus rotundus* and *Diaemus youngi* sampled in different states in the northern region of the country. Short (474 to 828 bp) and large (> 1400 bp) sequences of the 18S rRNA gene were obtained from DNA samples extracted from the spleens of *D. rotundus*. Phylogenetic analysis placed the amplified sequences into three distinct clades: 'South American Marsupialia Group,' *'Tapirus terrestris* group,' and '*Theileri*a sensu stricto,' indicating a high diversity of piroplasmids within this group of animals and the possibility of transmission of these agents by blood feeding (Mello et al., 2023a). Additionally, a study by Mello et al. (2023b) using DNA extracted from liver fragments of *D. rotundus* and *Diphylla ecaudata*, sampled across various Brazilian states, yielded negative results for the agents in question, likely due to the type of sample used (Mello et al., 2023b). Similarly, Piroplasmida DNA was not detected in spleen tissues from non-hematophagous bats sampled in the state of Acre, northern Brazil (Silva et al., 2025).

## Order Didelphimorphia

### Family Didelphidae

The first record of piroplasmids in marsupials in Brazil was made in 1961, based on blood smears from *Didelphis marsupialis* (common opossum) sampled in the state of Pará (Deane & Deane, 1961). Later, Serra-Freire (1979) identified evolutionary forms of *Babesia brasiliensis*, initially described as *Babesia ernestoi*, in blood smears from *Didelphis marsupialis* and *Didelphis albiventris* (white-eared opossum) sampled in Rio de Janeiro.

The detection of piroplasmids through PCR has been reported in specimens of *Monodelphis domestica* (gray short-tailed opossum), *Didelphis albiventris*, *Didelphis marsupialis*, and *Didelphis aurita* (black-eared opossum), sampled in the Federal District, Goiás, Maranhão, Mato Grosso, Mato Grosso do Sul, Minas Gerais, Pará, and Rio de Janeiro. The molecular prevalence rates obtained ranged from 2.22% (1/45) to 50%, with the latter positivity based on only two sampled animals (Wolf et al., 2016; Soares et al., 2017; Colle et al., 2019; Fava et al., 2022; Gonçalves et al., 2021; Braga et al., 2023; Oliveira et al., 2023).

A specimen of *Didelphis aurita*, sampled by Oliveira et al. (2023) and found positive for piroplasmids through PCR, exhibited clinical signs such as lethargy, weight loss, jaundice, pale mucous membranes, and anemia, which were confirmed by laboratory tests. These clinical and hematological signs were similar to those previously described in *Didelphis marsupialis* with high parasitemia for *B. ernestoi* (Serra-Freire, 1979).

Based the analysis of the nearly complete 18S rRNA gene sequences and partial sequences of other genetic markers (*cox-1* and *cox-3*) amplified from blood samples of *Didelphis albiventris* from the Federal District and Mato Grosso do Sul, and *Didelphis aurita* from Rio de Janeiro, a new phylogenetic clade was described and named as 'South American Marsupialia group.' While no species has been formally named, it is believed to belong to the genus *Babesia* (Gonçalves et al., 2021; Oliveira et al., 2023).

In a study conducted by Braga et al. (2023), a short sequence of the 18S rRNA gene was obtained from a blood sample of a *Didelphis marsupialis* specimen (2.2%, 1/45) captured in the state of Maranhão. The sequence was placed within the 'South American Marsupialia group' clade, providing evidence for the existence of a new species of *Babesia* in animals of the genus *Didelphis*. Recently, this putative new species was also detected in *Didelphis albiventris* in Argentina (Sebastian et al., 2025).

## Order Perissodactyla

### Family Tapiridae

A tapir (*Tapirus terrestris*) rescued in Campo Grande (MS) presented with a fracture in the left pelvic limb, apathy, hyperthermia, and anorexia. Laboratory tests revealed normocytic normochromic anemia, leukocytosis, neutrophilia, lymphocytosis, anisocytosis, and inclusions in erythrocytes suggestive of piroplasmids in the blood smear. Piroplasmid parasitism was confirmed by PCR, with the obtained sequence (414 bp) showing 98% identity with *T. equi* in a BLASTn analysis. Due to the presence of a fracture and osteomyelitis, it was not possible to determine whether the clinical and laboratory findings were related to infection by the agent (Silveira et al., 2017).

Subsequently, tapirs kept in captivity and sampled in the states of Amazonas and Pará tested positive for piroplasmids (57.9%; 11/19) by PCR based on the 18S rRNA gene. The amplified and sequenced fragments ranged from 392 to 475 bp (Gonçalves et al., 2020). Furthermore, Silva et al. (2021) reported a 35.3% positivity (6/17) for piroplasmids in spleen and blood samples from *Tapirus terrestris* that had been hit by vehicles in Mato Grosso. Two 776 bp sequences were placed in the same clade as sequences of *Theileria* sp. previously detected in cats in Brazil, a clade closely related to the *T. equi* clade. Additionally, Fava et al. (2022) detected piroplasmid DNA in a tapir (1/2 [50%]). The 18S rRNA sequence (553 bp) obtained was placed in a separate clade, but was phylogenetically related to *T. equi*.

In a study conducted with blood samples from wild tapirs in the state of Mato Grosso do Sul, 56.7% (56/99) tested positive for piroplasmids. Phylogenetic analyses, based on the nearly complete sequence of the 18S rRNA gene and additional sequences from the *cox-1* and *hsp70* genes, along with the identification of merozoites arranged in a 'Maltese Cross' formation in erythrocytes, led to the description of a new species, *Theileria terrestris*. In the phylogenetic tree, the obtained sequences were placed in a new clade, named the *'Tapirus terrestris* group,' which is phylogenetically related to the 'Equus group' clade (Mongruel et al., 2022).

Recently, four tapirs sampled in the state of Goiás tested positive for piroplasmids. Sequences of the near-complete 18S rRNA gene and *hsp70* obtained from two of these animals confirmed the presence of *Theileria terrestris* (Bittencourt et al., 2025).

## Order Primates

There are no specific studies focused on the detection of piroplasmids in non-human primates in Brazil. However, two studies aimed at detecting hemoparasites in different groups of captive animals sampled in Goiás and Minas Gerais reported positivity in PCR assays for piroplasmids in these animals, though no sequences were obtained (Fava et al., 2022; Castillo et al., 2024).

In the study by Fava et al. (2022), conducted on animals kept in rehabilitation centers in the states of Goiás and Minas Gerais, 28 non-human primates from the species *Alouatta caraya* (black howler) and *Callithrix penicillata* (black-tufted marmoset) were sampled. Of these, five (17.5%) tested positive for piroplasmids by PCR, and 13 (46.4%) showed intracellular inclusions suggestive of these agents in blood smears. Furthermore, Castillo et al. (2024) reported a 22.2% (4/18) positivity in PCR for piroplasmids in non-human primates sampled in the state of Minas Gerais. The positive animals included representatives of *Ateles* sp. (spider monkey), *Sapajus apella* (Black-capped Capuchin), and *Gorilla gorilla* (gorilla).

## Order Rodentia

### Family Caviidae

Criado-Fornelio et al. (2009) reported a molecular occurrence of 14.3% (2/14) for piroplasmids in capybaras (*Hydrochoerus hydrochaeris*) sampled in Pelotas, Rio Grande do Sul. The 1714 bp sequence of the 18S rRNA gene obtained was placed in a clade sister to the '*Babesia* sensu stricto' clade and was named *Babesia* sp. capybara. Later, a molecular prevalence rate of 39.3% (11/28) for this agent was reported in a study conducted in the state of Goiás. In this study, large sequences of the 18S rRNA gene (1519 - 1561 bp) were amplified, which grouped into a unique clade along with the previously cited sequence. Additionally, sequences from the *hsp70* and *cox-3* genes were also amplified, and these were placed in a separate clade in the phylogenetic trees. As a result, a new species, *Babesia goianiaensis*, was described, and another clade, the 'Capybara group,' was added to the phylogeny of piroplasmids (Krawczak et al., 2023; Neves et al., 2023). Piroplasmid DNA was detected in two capybaras (50% [2/4]) also sampled in the state of Goiás. A 565 bp sequence obtained from one of the animals showed 100% identity with *B. goianiaensis* (Bittencourt et al., 2025).

Additionally, short fragments of the 18S rRNA gene were amplified from DNA samples extracted from nymphs and adults of *Amblyomma dubitatum* collected from capybaras (Gonçalves et al., 2021; Neves et al., 2023), *Rattus rattus* (Gonçalves et al., 2021), and coatis (Perles et al., 2023) sampled in the states of Mato Grosso do Sul and Goiás, as well as from specimens of *Amblyomma sculptum* collected from capybaras (Neves et al., 2023; Krawczak et al., 2023) and coatis (Perles et al., 2023) in the same states. These sequences were phylogenetically associated with *Babesia* sp. detected in capybaras, suggesting that *A. dubitatum* and *A. sculptum* may act as vectors for this agent. However, vector competence experiments should be conducted to confirm this hypothesis (Neves et al., 2023; Krawczak et al., 2023).

### Family Cuniculidae

Four peccaries - *Cuniculus paca* (12.1% [4/33]) sampled in the state of Mato Grosso tested positive for piroplasmids based on PCR targeting the 18S rRNA gene. The 537 bp sequence was placed in a clade phylogenetically associated with *Babesia* sp. capybara (Soares et al., 2017).

### Family Dasyproctidae

A single agouti (*Dasyprocta* sp.) sampled in the state of Mato Grosso tested positive for piroplasmids by PCR. The obtained sequence (314 bp) of the 18S rRNA gene was placed in a clade close to sequences of *Theileria* sp. detected in *Dasypus novemcinctus* (nine-banded armadillo) sampled in the same study. This clade was positioned near *Theileria* sensu stricto (Soares et al., 2017).

### Family Echimyidae

The first study to molecularly detect piroplasmids in wild rodents in Brazil was conducted by Wolf et al. (2016) on animals sampled in the Pantanal of Poconé, state of Mato Grosso. Out of 11 *Trichomys pachyurus* captured, three (27.3%) tested positive for the agents under study. The 532 bp sequence obtained from the amplification of the 18S rRNA gene was placed in a clade containing a sequence of *Babesia* sp. detected in *M. domestica* from the same study.

Subsequently, Sousa et al. (2018) detected piroplasmid DNA in six *Trichomys fosteri* (7.8% [6/110]) in the Pantanal of the state of Mato Grosso do Sul. Five of the obtained sequences (approximately 700 bp) were placed in a clade together with *B. vogeli*. In contrast, one sequence was grouped with sequences of *T. equi*.

A recent study conducted in the state of Mato Grosso reported a positivity rate of 11.7% (17/145) for piroplasmids in captured rodents. All positive samples came from *Trichomys pachyurus*. Large sequences of the 18S rRNA gene (1200 - 1230 bp) and sequences between 700 and 800 bp of the *cytB* gene were obtained. In both phylogenetic inferences, the sequences clustered into a single clade. In the 18S rRNA-based phylogeny, the sequences were positioned in a clade sister to the 'Phyllostomidae bat group.' This new clade was named the 'South American Rodentia group' (Pacheco et al., 2025).

Additionally, other studies reported the presence of piroplasmid evolutionary forms in blood smears of rodents from the Cricetidae family (*Oligoryzomys nigripes*, *Akodon montanensis*, *Delomys sublineatus*, and *Nectomys squamipes*) and the Muridae family (*Rattus norvegicus*) sampled in Rio de Janeiro (Gazeta et al., 2004; Silva et al., 2007).

### Family Erethizontidae

Piroplasmids were detected by PCR in *Coendou prehensilis* (Brazilian porcupine) (70% [7/10]) and *Sphiggurus spinosus* (Paraguayan hairy dwarf porcupine) (100% [1/1]) sampled at rehabilitation centers in the states of Goiás and Minas Gerais. However, no sequences were obtained (Fava et al., 2022). Subsequently, one Brazilian porcupine (16.7% [1/6]) sampled in Goiás tested positive for these agents, and a 304 bp fragment of the 18S rRNA gene showed 100% identity with *B. vogeli* previously detected in a dog from Brazil (Bittencourt et al., 2025). All abovementioned studies used blood samples for Piroplasmida detection.

## Superorder Xenarthra (Orders Cingulata and Pilosa)

The Superorder Xenarthra comprises two orders: Cingulata (which includes armadillos) and Pilosa (which includes anteaters and sloths). In Brazil, the first description of piroplasmids in these animals was made by Lainson et al. (1979), who found evolutionary forms of *Theileria* sp. in blood smears from *Dasypus novemcinctus* (nine-banded armadillo). Later, Soares et al. (2017) detected a new genotype of the 18S rRNA gene (473 bp) in *D. novemcinctus* (9.37% [3/32]) sampled in the state of Mato Grosso. This new genotype clustered in a separate clade from the others, closely related to *Theileria* sp. detected in *Dasyprocta* sampled in the same study (Soares et al., 2017).

Additionally, a 12.5% (4/32) positivity rate for piroplasmids was detected by PCR targeting the 18S rRNA gene in biological samples from *Myrmecophaga tridactyla* (giant anteater) and *Tamandua tetradactyla* (Southern Tamandua) collected from animals in rehabilitation centers in the states of Goiás and Minas Gerais. However, it was not possible to obtain sequences from the amplified samples (Fava et al., 2022).

In a study by Calchi et al. (2023), a molecular prevalence rate of 5.5% (25/455) for piroplasmids was found in animals belonging to the superorder Xenarthra, including giant anteaters, Southern Tamandua, giant armadillos (Priodontes maximus), nine-banded armadillos, six-banded armadillos (*Euphractus sexcinctus*). The short sequences obtained from the 18S rRNA gene were positioned in different clades in the phylogenetic analysis. Piroplasmida sequences from *P. maximus* clustered within the 'South American Marsupialia group' clade, while the sequence from *D. novemcinctus* grouped with a previously obtained sequence from the same species, forming a sister clade to *Theileria* sensu stricto. These findings suggest that xenarthrans may be parasitized by both *Babesia* spp. and *Theileria* spp. Furthermore, ITS-1 sequences obtained from anteater DNA formed a distinct clade, supporting the presence of a diverse array of these protozoa in Xenarthra.

Recently, two *M. tridactyla* individuals (9.5% [2/21]) sampled in the state of Goiás tested positive for piroplasmids through amplification of the 18S rRNA gene; however, no sequences were obtained (Bittencourt et al., 2025).

## Birds

Few studies have investigated the occurrence of piroplasmids in wild birds in Brazil. In this context, Quillfeldt et al. (2014) reported the presence of *Babesia* spp. DNA, phylogenetically related to *Babesia poelea* (clade 'Percei group' – 18S rRNA gene), in brown boobies (*Sula leucogaster*) (7% [12/172]) and masked boobies (*Sula dactylatra*) (13.1% [22/168]) sampled on islands along the Brazilian coast, including the Abrolhos Archipelago, Fernando de Noronha, Rocas Atoll, and the São Pedro and São Paulo Archipelago.

Six Orinoco geese (*Neochen jubata*) (9.68% [6/62]) sampled in the state of Goiás tested positive for piroplasmids. One of the positive samples was sequenced (693 bp fragment of the 18S rRNA gene) and was phylogenetically grouped within the *B. vogeli* clade (Werther et al., 2017).

In a study conducted on a beach in Florianópolis, Santa Catarina, only one Atlantic yellow-nosed albatross (*Thalassarche chlororhynchos*) out of 144 sampled seabirds tested positive for piroplasmids (0.69%). The obtained sequence (672 bp of the 18S rRNA gene) clustered within the *Babesia* sensu stricto clade, alongside sequences of *Babesia kiwiensis* previously detected in *Apteryx australis mantelli* (North Island brown kiwi) and *Babesia* spp. identified in *Turdus falcklandii* (Austral thrush) and *Morus serrator* (Australian gannet) (Sgarioni et al., 2023). Additionally, Procellariiformes (n = 52) and Magellanic penguins (*Spheniscus magellanicus*) (n = 194) sampled in coastal regions of the states of São Paulo and Santa Catarina tested negative for piroplasmids (Machado et al., 2025).

## Exotic Animals

The first report of fatal cytauxzoonosis in Brazil involving exotic animals was described in captive lions (*Panthera leo*) by Peixoto et al. (2007). A lion cub died suddenly, and approximately 40 days later, the mother lioness began exhibiting clinical signs, including weight loss, apathy, anemia, pale mucous membranes, alopecia, dark-colored urine, tachypnea, nystagmus, motor incoordination, deafness, and nasal and ocular discharge. These signs progressed until her eventual death. Laboratory findings revealed anemia, neutrophilia, monocytosis, lymphopenia, eosinopenia, thrombocytopenia, and elevated blood urea levels. Urinalysis showed pyuria, hematuria, and proteinuria. Necropsies of both animals demonstrated the presence of intra-histiocytic schizonts obstructing blood vessels in various organs, a pathognomonic lesion of cytauxzoonosis (Peixoto et al., 2007).

Additionally, André et al. (2011) detected *Babesia* sp. DNA in a common genet (*Genetta genetta*) housed in a zoo in the state of São Paulo. A 526 bp fragment of the 18S rRNA gene was amplified and sequenced, revealing 99% identity with *B. leo* previously detected in a domestic cat from South Africa.

## Phylogeny and Lineages of Piroplasmids

**Figure 2:**
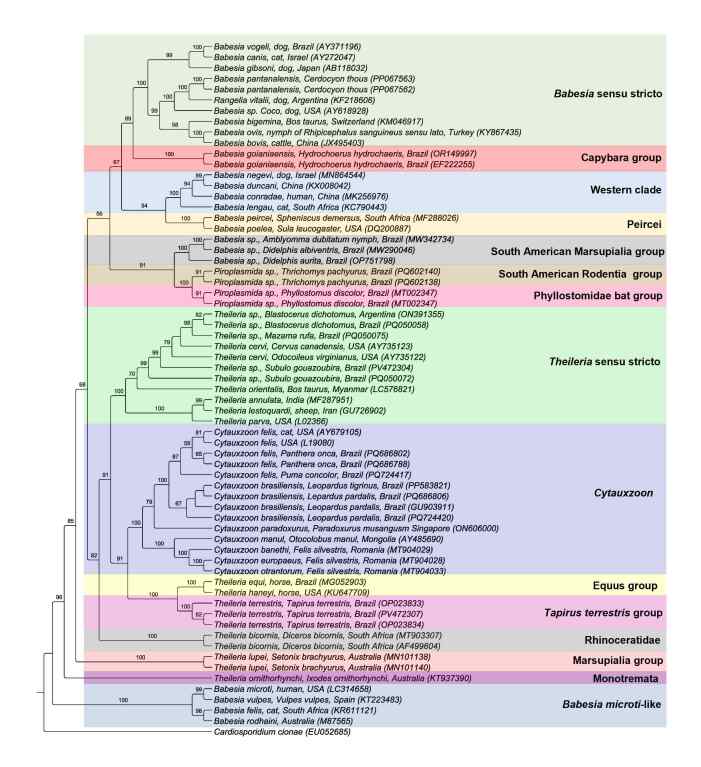
Maximum likelihood phylogenetic analysis based on a 1,726 characters alignment of 18S rRNA gene sequences from piroplasmids, using the TIM3+I+G evolutionary model. *Cardiosporidium cionae* was used as the outgroup.

Piroplasmids constitute a polyphyletic group. A previous phylogenetic study based on the 18S rRNA gene, conducted by Jalovecka et al. (2019), identified ten distinct clades, namely: (I) *Babesia microti*-like, composed of *Babesia rhodaini* (infects rodents), *Babesia felis* (infects felines), *Babesia vulpes* (canids and mustelids), *Babesia microti* (rodents and primates); (II) Monotremata (*Theileria ornythorhynchi*); (III) Western Clade (*Babesia duncani*, *Babesia lengau*, *Babesia conradae*, and *Babesia negevi*) that can affect the families Bovidae, Cervidae, Canidae, Felidae, Herpestidae, and humans; (IV) Peircei (*Babesia peircei*, *Babesia poelea*, and *Babesia ugwidiensis*) that infect birds; (V) Marsupialia, composed of *Theileria* spp. that affect marsupials from Australia; (VI) Rhinocerotidae (*Theileria bicornis*); (VII) *Cytauxzoon* spp. (*C. felis*, *Cytauxzoon manul*, *Cytauxzoon otrantorum*, *Cytauxzoon banethi*, *Cytauxzoon europaeus*, and *C. brasiliensis*) that occur in animals from the Felidae, Ursidae, and Herpestidae families; (VIII) Equus (*T. equi* and *Theileria haneyi*) that affect equids; (IX) *Theileria* sensu stricto (*Theileria annulata*, *Theileria parva*, *Theileria lestoquardi*, and *Theileria orientalis*) that affect the Bovidae and Cervidae families; and group (X) *Babesia* sensu stricto (*B. bovis*, *B. bigemina*, *B. canis*, and *Babesia ovis*) described in several species of animals (Marsupialia, Bovidae, Cervidae, Giraffidae, Rodentia, Canidae, Mustelidae, Ursidae, Felidae, birds, and humans). *Rangelia vitalli*, a piroplasmid found in domestic and wild canids in South America (Brazil, Argentina, and Uruguay), is placed in the *Babesia* sensu stricto clade (Soares et al., 2011; Lemos et al., 2012; Eiras et al., 2014; Soares, 2014; Soares et al., 2015) (**Figure 2**).

Based on studies conducted in Brazil, five new clades have been described and added to the phylogeny: (I) Phyllostomidae group, composed of piroplasmids detected in non-hematophagous bats of the Phyllostomidae family (Ikeda et al., 2021); (II) South American Marsupialia group, composed of *Babesia* spp. detected in opossums (*Didelphis* spp.) and *A. dubitatum* (Gonçalves et al., 2021; Oliveira et al., 2023; Braga et al., 2023); (III) Capybara group, composed of *B. goianiaensis*, described in capybaras (Krawczak et al., 2023); (IV) *T. terrestris* group formed by *T. terrestris*, described in tapirs (Mongruel et al., 2022); and (V) South American Rodentia group, which includes *Piroplasmida* spp. detected in rodents of the species *T. pachyurus* (Pacheco et al., 2025) (**Figure 2**).

This study presents, in **Figure 2**, a phylogeny encompassing all Piroplasmida phylogenetic clades described to date. For this purpose, 18S rRNA sequences of piroplasmid species were selected, representing the main species of each clade deposited in GenBank. The sequences were subsequently aligned using MAFFT software (Katoh et al., 2019) and trimmed with Bioedit v. 7.0.5.3 (Hall, 1999). The W-IQ-Tree software was employed to choose the evolutionary model based on the BIC criterion (TIM3+I+G) and for phylogenetic analysis using the Maximum Likelihood method (Trifinopoulos et al., 2016). The phylogenetic trees were edited using Treegraph 2.0.56-381 beta software (Stöver & Müller, 2010).

## Final Remarks

The extensive genetic diversity of piroplasmids infecting a wide range of wild animals and distributed across nearly the entire country, as demonstrated in this study, underscores the complexity of Brazilian ecosystems and highlights the importance of continued research into the interactions among pathogens, hosts, and the environment. Ongoing investigation in this field is crucial for enhancing wildlife conservation and disease management strategies, thereby promoting ecological balance and safeguarding the health of both wildlife and human populations.

Therefore, further studies are needed to: (i) identify the potential vectors of the piroplasmid species circulating in the country; (ii) investigate the role of wild animals in maintaining these agents; (iii) amplify large fragments of the 18S rRNA gene and other genetic markers to improve species identification and discrimination; (iv) sequence the mitogenomes of these organisms to perform phylogenetic inferences (Schreeg et al., 2016), thereby enhancing the understanding of their evolution and facilitating the description of new species; (v) employ advanced techniques, such as metagenomics, single-cell analysis, and hybrid assemblies, which will enable the assembly of genomes from these agents that are challenging to culture (Schoenle et al., 2025). It is also essential to conduct surveys in other regions of the country and in additional animal species to enhance our understanding of the diversity of these agents, their potential impact on wildlife conservation, spill over to domestic animals and the possible zoonotic risk associated with these agents.

## Data Availability

Data are contained within the article.
